# Enterovirus 71 Infection Causes Severe Pulmonary Lesions in Gerbils, *Meriones unguiculatus*, Which Can Be Prevented by Passive Immunization with Specific Antisera

**DOI:** 10.1371/journal.pone.0119173

**Published:** 2015-03-13

**Authors:** Fang Xu, Ping-Ping Yao, Yong Xia, Lei Qian, Zhang-Nv Yang, Rong-Hui Xie, Yi-Sheng Sun, Hang-Jing Lu, Zi-Ping Miao, Chan Li, Xiao Li, Wei-Feng Liang, Xiao-Xiao Huang, Shi-Chang Xia, Zhi-Ping Chen, Jian-Min Jiang, Yan-Jun Zhang, Ling-Ling Mei, She-Lan Liu, Hua Gu, Zhi-Yao Xu, Xiao-Fei Fu, Zhi-Yong Zhu, Han-Ping Zhu

**Affiliations:** 1 Key Lab of Vaccine against Hemorrhagic Fever with Renal Syndrome, Zhejiang Province Center for Disease Prevention and Control, Hangzhou, China; 2 Department of Pathology, First Municipal Hospital of Hangzhou, Hangzhou, China; 3 The First Affiliated Hospital, Zhejiang University, Hangzhou, China; 4 Hangzhou Sixth People’s Hospital, Hangzhou, China; 5 Wenzhou Medical University, Wenzhou, China; 6 Jiaxing Center for Disease Control and Prevention, Jiaxing, China; Indiana University, UNITED STATES

## Abstract

Neurogenic pulmonary edema caused by severe brainstem encephalitis is the leading cause of death in young children infected by Enterovirus 71 (EV71). However, no pulmonary lesions have been found in EV71-infected transgenic or non-transgenic mouse models. Development of a suitable animal model is important for studying EV71 pathogenesis and assessing effect of therapeutic approaches. We had found neurological disorders in EV71-induced young gerbils previously. Here, we report severe pulmonary lesions characterized with pulmonary congestion and hemorrhage in a gerbil model for EV71 infection. In the EV71-infected gerbils, six 21-day-old or younger gerbils presented with a sudden onset of symptoms and rapid illness progression after inoculation with 1×10^5.5^ TCID_50_ of EV71 via intraperitoneal (IP) or intramuscular (IM) route. Respiratory symptoms were observed along with interstitial pneumonia, pulmonary congestion and extensive lung hemorrhage could be detected in the lung tissues by histopathological examination. EV71 viral titer was found to be peak at late stages of infection. EV71-induced pulmonary lesions, together with severe neurological disorders were also observed in gerbils, accurately mimicking the disease process in EV71-infected patients. Passive transfer with immune sera from EV71 infected adult gerbils with a neutralizing antibody (GMT=89) prevented severe pulmonary lesion formation after lethal EV71 challenge. These results establish this gerbil model as a useful platform for studying the pathogenesis of EV71-induced pulmonary lesions, immunotherapy and antiviral drugs.

## Introduction

Enterovirus 71 (EV71), a member of the genus *Enterovirus* within the family *Picornavirus*, affects mostly infants and young children [1–3]. EV71 infection usually leads to hand, foot, and mouth disease (HFMD) and sometimes causes severe neurological manifestations including aseptic meningitis, encephalitis, acute flaccid paralysis, and pulmonary edema, with the percentage of pulmonary edema or hemorrhage considerably high in the fatal cases [4,5]. EV71-infection, responsible for severe nervous system damage and death, is now considered the most important neurotropic enterovirus following the eradication of poliomyelitis [6].

In 2010 alone, at least 1.7 million HFMD cases were reported with 905 deaths [7]. Between 2008 and 2012, there were 7.2 million probable HFMD cases and a potential 2,457 EV71-related deaths reported to Chinese Center for Disease Control and Prevention [8]. Although EV71 outbreaks have occurred sporadically in several geographies, several large outbreaks have occurred in Asia-Pacific region including mainland China[9–11], Taiwan[4], South Korea[12], Japan[13], Singapore[14], Malaysia[15], Thailand, and Vietnam[16]. The 1998 Taiwanese outbreak resulted in 65 HFMD deaths of EV71-infected children, mainly due to pulmonary edema or hemorrhage[4]. EV71 was confirmed as the major pathogen in the 2008 Chinese outbreak that resulted in 4.89 million HFMD cases and 126 deaths [10].

Children infected with EV71 tend to have a faster disease progression, higher fever and a higher incidence of limb movement disorder, coma, neurological damage, neurogenic pulmonary edema and death compared to other enterovirus strains [17]. However, the pathogenesis of EV71 infection is not clearly elucidated. Though several mouse species have been used to study EV71 infection and pathogenesis (BALB/c, ICR, NOD/SCID, AG129, and transgenic strains), no models were successful in inducing pulmonary edema [18–25]. In previous studies, we demonstrated that gerbils infected with EV71 developed symptoms related to neurological lesions including hind limb paralysis, slowness, ataxia and lethargy [26]. In this report, we found that gerbils aged 7 to 21 days infected with EV71 by intraperitoneal (IP) or intramuscular (IM) routes and exhibited severe pulmonary lesions. We also demonstrated that EV71-induced pulmonary lesions could be prevented by passive transfer of specific EV71-antisera after lethal EV71 challenge. Therefore, the gerbil EV71 model may serve as a useful animal model for studying the pathogenesis of EV71-mediated pulmonary disease and assessing novel therapeutic interventions.

## Materials and Methods

### Virus

The EV71 strain used in this study (58301, genotype C4) was isolated from a vesicle swab obtained from a 12-month-old child with mild HFMD in 2008 in Hangzhou, China [26]. The child’s parents provided written informed consent for the scientific use of the sample, and Hangzhou Sixth People’s Hospital Ethics Committee approved the project. EV71 was grown in the Vero cells and passaged no more than five generations before use. EV71 was stored in aliquots at -70°C in modified Eagle’s medium (MEM) supplemented with 10% fetal bovine serum (FBS). Virus titer was 1×10^7.0^ tissue culture infection dose (TCID_50_) determined using Vero cells according to the Reed—Muench method [27].

### Animals and EV71 infection

Gerbils were purchased from the Animal Center of Zhejiang Academy of Medical Sciences, Hangzhou, China. The animals were housed under standard laboratory conditions (relative humidity 50±5%, room temperature 24±1°C, and 12 hrs light dark cycle) in Plexiglas cages (Techniplast, Italy; 1291H). The animals were fed with a standard diet and had unlimited access to water. All animal experimental procedures were performed following the Regulations for the Administration of Affairs Concerning Experimental Animals of the People’s Republic of China, and were approved by Institutional Animal Care and Use Committee of Zhejiang Provincial Center for Disease Control and Prevention.

Gerbils were inoculated with EV71 via either an IP, IM, or intracranial (IC) route, and clinical scores were defined as following: 0, healthy; 1, ruffled hair, hunchbacked or reduced mobility; 2, limb weakness; 3, paralysis in one limb; 4, paralysis in both limbs or deep lethargy; 5, death. When infected-gerbils showed grade 4 symptoms, gerbils were humanely sacrificed. While gerbils were most susceptible to EV71 infection via IC, this usually led to mechanical injury and unnatural death (data not shown). While IP and IM routes had similar infection rates in gerbils, the IP route was chosen as the primary inoculation route for its convenience.

Four sets of animal experiments were performed. In the first study, gerbils were inoculated via the IP route with 1×10^5.5^ TCID_50_ of EV71 at 7, 14, 21, 28 and 35 days (n = 6 for each age group). In the second study, 21-day-old gerbils were inoculated with 1×10^3.5^ or 1×10^5.5^ TCID_50_ of EV71 via the IP route (n = 8 for each dose group). In the third study, 21-day-old gerbils were inoculated with 1×10^5.5^ TCID_50_ of EV71 via IP, IM or oral (OL) routes respectively. Eight gerbils were used for each different infection routes. These gerbils were observed daily for 20 days after inoculation. When infected-gerbils showed grade 4 symptoms, gerbils were euthanized and tissues were collected for histological examination. In the fourth study, three 50-day-old gerbils were administered EV71 via the IP route with 1×10^5.5^ TCID_50_ of EV71, blood samples were collected at 0, 7, 14, 21, 28 and 35 days post infection (p.i.) for EV71 neutralizing antibody analysis.

### Lung virus titer of infected gerbils

Lung tissues were collected after signs of lethargy or 2 hind limb paralysis appeared in EV71-infected gerbils at the age of 7, 14, 21, 28, and 35 d. After perfusion with 0.02 M PBS (pH = 7.2), lung tissue samples were aseptically removed, weighed, and stored at -70°C. The tissue samples were homogenized in PBS to generate a 10% solution followed by freezing and thawing three times. The tissue suspension was centrifuged at 1000 x *g* for 5 min at 4°C to remove tissue debris. The supernatants were serially diluted in MEM, and 100 μL of each dilution were placed onto monolayer of Vero cells in 96-well plates for virus titration. Following 4-day incubation at 37°C, the plates were scored for cytopathic effects (CPE) positive wells microscopically and the TCID_50_ was determined by the highest diluted titers and expressed as log TCID_50_ / g of tissue.

### Histological examination

Lung tissues from the gerbils exhibiting clinical symptoms (approximately 4–5 day p.i.) were fixed in 10% formalin in PBS for 48 h and embedded in paraffin. The paraffin-embedded tissue sections were mounted on poly-L-lysine-coated slides, and stained with hematoxylin and eosin for morphological examination as described previously [26].

### Quantitative RT-PCR

Tissues from gerbils were homogenized and total RNA was prepared using the RNeasy Mini kit (Qiagen, USA) according to the manufacturer’s instructions. The extracted RNA was analyzed for the viral load using the TaqMan quantitative RT-PCR for amplification of the EV71 VP1 gene as described previously [26]. Each assay was performed in triplicate. The standard curve was created by 10-fold serial dilutions of stock EV71 (1×10^7.0^ TCID_50_/ mL).

### Detection of antibodies against EV71

Blood samples were collected from 50-day-old gerbils on days 0, 5, 7, 14, 21, 28, and 35 post-EV71 inoculation. EV71 neutralizing antibodies were analyzed using a standard protocol. Briefly, two-fold dilutions of heat-inactivated sera were mixed with 50 μL EV71-containing solution at a dose of 1×10^2.0^ TCID_50_ per well in a 96-well plate, and incubated for 2 h at 37°C. After incubation, mixtures were added onto monolayer of Vero cells and the cells were inspected daily for CPE up to 4 d. Neutralizing antibody titers were determined as the highest dilution of serum that inhibited virus growth. Fluorescent antibodies to EV71 were identified using an indirect immunofluorescence assay (IFA). Briefly, 10 μL of two-fold serially diluted sera were applied to each well of the slide containing EV71-infected Vero cells fixed in acetone and incubated for 30 min at 37°C. Following washing in 1X PBS three times for 10 min, slides were combined with 10 μL fluorescein isothiocyanate (FITC)-labeled anti-mouse IgG (Sigma) at 37°C for 30 min. The slides were washed as before, covered with cover slips, and fluorescence was examined under a fluorescent microscope (Leica DMI 4000B, Leica Microsystems, Wetzlar, Germany).

### Passive immunization

Adult gerbils were immunized with formalin-inactivated EV71 and boosted 1 week later [26]. Serum samples were collected from the immunized gerbils 1 week post-boosting dose and following the same time course for the mock-immune gerbils. Heat-inactivated (56°C for 30 min) sera were tested for neutralizing antibodies against EV71 at dilutions up to 1:256. The 21-day-old treatment group was passively immunized by IP with 100 μL immune sera and challenged with IP injection 1 h later with 100×HD_50_ (humane endpoint) of EV71. A second dose of immune sera was administered 24 h post-challenge. Twenty-one-day-old gerbils (n = 5) from control groups were given mock-immune sera. Gerbils were monitored for 20 days post-challenge for clinical symptoms.

### Statistical analysis

All statistical analyses were done with GraphPad Prism, version 5.0 (GraphPad 4 Software, San Diego, CA). The survival rates of gerbils infected with EV71 were analyzed by log-rank analysis. Clinical scores were analyzed using the Wilcoxon test. Viral copies in tissues after EV71 infection were expressed as mean ± SD and analyzed with a Student’s *t* test by SPSS software. A P value less than or equal to 0.05 was considered statistically significant.

## Results

### Clinical symptoms after EV71 infection

**Fig 1:**
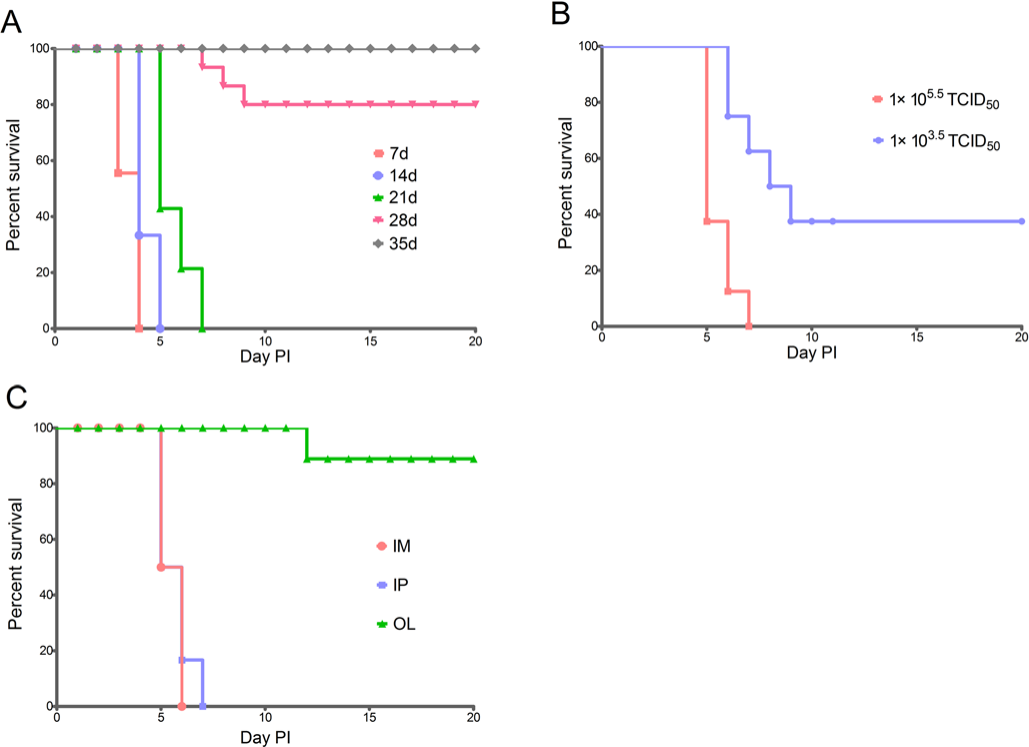
Survival rates of gerbils infected with EV71. (A) Gerbils (7, 14, 21, 28, and 35 d old) were inoculated IP with 1x10^5.5^ TCID_50_ of EV71 (n = 6 for each age group). (B) 21-day-old gerbils were inoculated with 1×10^3.5^ or 1×10^5.5^ TCID_50_ of EV71 via IP (n = 8 for each dose group). (C) 21-day-old gerbils were inoculated with 1×10^5.5^ TCID_50_ of EV71 via IP, IM or OL respectively.

In order to examine the susceptibility of gerbils at different ages to EV71 infection, three groups of gerbils at the age of 7, 14 and 21 days were IP inoculated with 1×10^5.5^ TCID_50_ of EV71 (n = 6 for each age groups). All gerbils presented with a sudden onset of symptoms at 3–4 days post-inoculation. In addition, all gerbils showed disease signs including progressing weakness, one or two hind limb paralysis and deep lethargy. Illness progressed rapidly and usually caused death within 12 hours of onset. Ten gerbils (two in 7 days, four in 14 days and four in 21 days of age) also had respiratory system symptoms including tachypnea, respiratory distress, apnea, or rhythm changes. Clinical signs between the three age groups were not different, with the only variation of the time from EV71 inoculation to disease onset. The average time of disease onset and death was 3 and 3.7 p.i., respectively, for gerbils in the 7-day-old group. For gerbils in the 14-day-old or 21-day-old groups, the average time of disease onset was 4–5 days and death occurred within 6–12 h after clinical signs occurred. In the 28-day-old group, two of out six gerbils began to exhibit hind limb weakness and paralysis five days post-IP inoculated with 1×10^5.5^ TCID_50_ of EV71 and died two days later. Four gerbils survived and one experienced hind limb paralysis. There was no mortality in the 35-day-old group with only two gerbils presenting with hind limb paralysis (Fig. 1A, S1–S5 Tables). These data demonstrated that gerbils aged 7–21 days were most sensitive to the EV71 infection.

Based on the above clinical findings, we further investigated the course of EV71 infection and disease progression in 21-day-old gerbils by IP inoculation at lower EV71 doses (1×10^3.5^ TCID_50_). Gerbils infected at these lower doses developed clinical signs later than higher dose group (1×10^5.5^ TCID_50_), with the average time from infection to symptoms onset equaling seven days. Furthermore, three of eight gerbils were asymptomatic after lower dose infection, while two were positive for anti-EV71 antibodies. Finally, the incidence of death was 62.5% (5/8) versus 100% in high titer infected animals (Fig. 1B, S6 Table). These data indicate that disease progress and death rate are dose dependent, which may relate to high virus replication level in the targeted organs of gerbils infected with higher doses of EV71.

To analyze the susceptibility of gerbils to EV71 by different injection routes, 24 21-day-old gerbils were inoculated with EV71 (1×10^5.5^ TCID_50_) by IM, IP, or OL route (n = 8 for each route). Disease onset was similar between IM and IP routes (4–5 day p.i), and all gerbils died by 5–7 days after inoculation. In contrast OL administration of EV71 led to a time of disease onset of 10 days p.i. and only one gerbil died during the experimental period (Fig. 1C, S7 Table). The HD_50_ for IM and IP inoculation were 1×10^3.0^ TCID_50_ and 1×10^2.64^ TCID_50_, respectively.

### Histopathological observations in lung tissues

**Fig 2:**
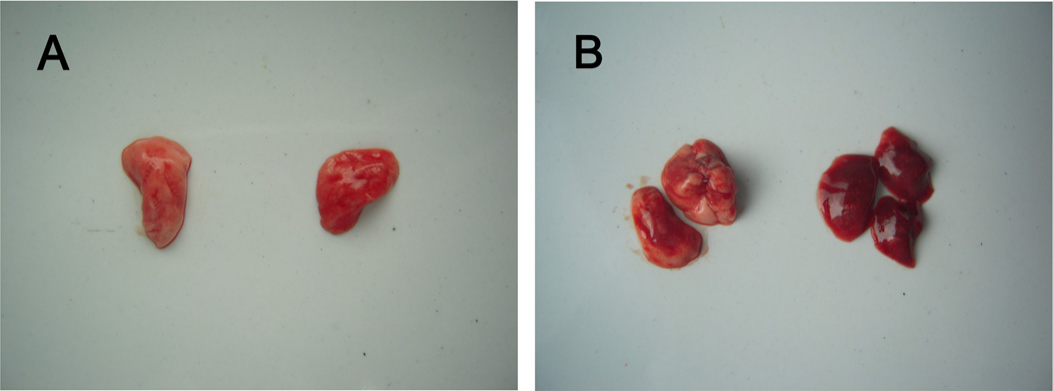
Macroscopic pathological lung changes in EV71-infected 21-day-old gerbils following 1×10^5.5^ TCID_50_ inoculation by IP route. Pictures correspond to the lung tissues from healthy or EV71-infected gerbils. Normal lung of a healthy gerbil (A, left; B, left), markedly red and congestion, extensive hemorrhage (A, right; B, right) in the lung were shown in EV71-infected gerbils.

**Fig 3:**
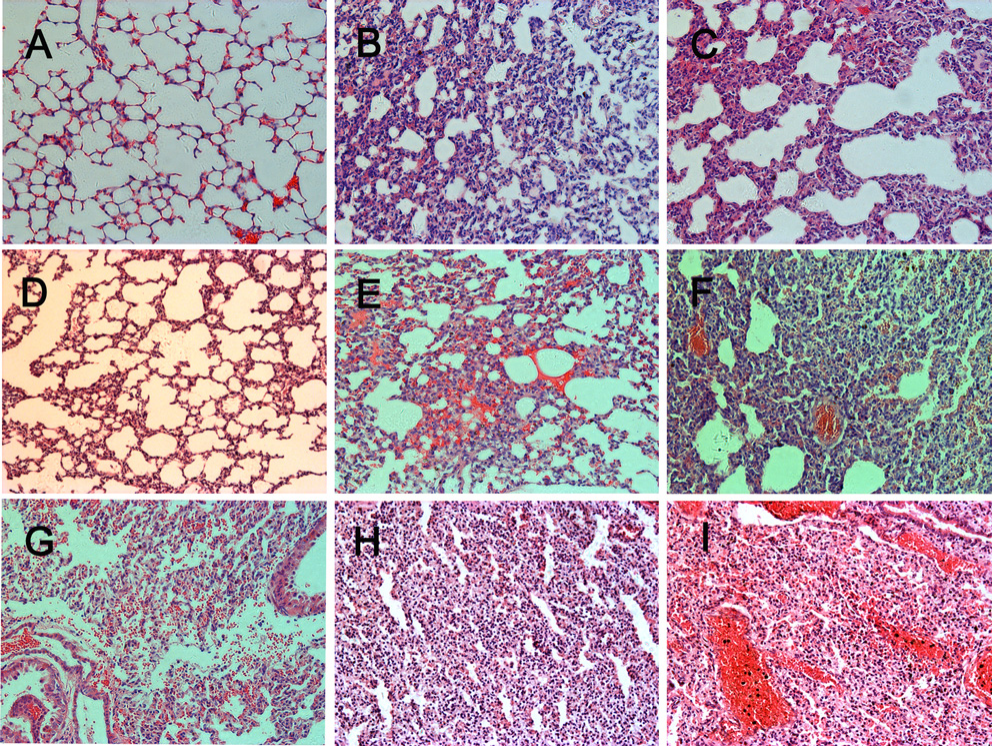
Serial pathological changes in lung tissues of gerbils infected with 1×10^5.5^ TCID_50_ EV71 by IP route. (A) Normal gerbil lung tissues. Typical lesions included multiple foci of pulmonary interstitial inflammatory (B) and alveolar septum broadening (C) was seen in lung at the age of 7–21 days of EV71-infected gerbils. Some fluid could be seen in alveolar space (D) and focal alveolar hemorrhage were noted (E). Local pulmonary consolidation (F) and the lumina of alveoli and bronchioles were filled with edema fluid and red cells (G). Extensive alveolar consolidation, pulmonary interstitial inflammatory cell infiltration (H), or extensive pulmonary hemorrhage and alveolar spaces filled with mononuclear and neutrophilic infiltration (I) were shown at the end stage of EV71-infection. Hematoxylin and eosin stain; magnification: A-I, 200x.

For gerbils, the spinal cord, brainstem and skeletal muscle are the target organs of EV71-infection [26]. Neuronal degeneration, neuronal loss and neuronophagia in central nervous system (CNS) and necrotizing myositis in skeletal muscle were observed in the infected gerbils [26]. In this study, severe damage in lung tissues were also found in infected gerbils. Lung tissue from EV71-infected gerbils was observed to be more dark red than that from normal gerbils (Fig. 2). Microscopically, lung tissue from healthy gerbils showed normal alveoli, alveolar septa, and lining epithelium (Fig. 3A). In contrast, lung tissues of EV71-infected gerbils revealed diffused lesions and dysfunctions with varying degrees of severity. Pulmonary interstitial inflammatory and alveolar septum broadening was observed in all EV71-infected gerbils after EV71 inoculation at both high IP dosing in all three age groups (1×10^5.5^ TCID_50_; 7 d, 14 d, 21d) (Fig. 3B). A thickened alveolar septum resulting from inflammation, scarring, or extra fluid (edema) was the main histological feature (Fig. 3C). Occasionally, lung tissue or alveolar space contained liquid instead of gas (Fig. 3D) and focal alveolar hemorrhaging was noted (Fig. 3E). One third of infected gerbils (6/18) developed focal alveolar consolidation and many neutrophilic infiltrates were found within the alveolar spaces (Fig. 3F). In addition, another one third developed diffuse alveolar hemorrhaging and lumina of alveoli with bronchioles minority filled with edema fluid (Fig. 3G). There was no obvious difference in histopathology in infected gerbils among the three age groups (Table 1). These data indicated gerbils aged from 7–21 days exhibited an age-independent response to lung tissue damage and death rate although the time to disease onset was different in each. Both 28-day-old and 35-day-old gerbils showed only pulmonary interstitial thickening, with no extensive pulmonary hemorrhaging observed (data not shown).

**Table 1:** Lung pathology following EV71 infection.

Pathological findings	7 d (n = 6)		14 d (n = 6)		21 d (n = 6)	
	n	%	n	%	n	%
Pulmonary interstitial inflammatory cell infiltration	6	100.0	6	100.0	6	100.0
Alveolar septum broadening	6	100.0	6	100.0	5	83.3
Local alveolar consolidation	2	33.3	1	16.7	3	50.0
Interstitial pulmonary congestion	6	100.0	6	100.0	5	83.3
Extensive pulmonary hemorrhage	2	33.3	1	16.7	3	50.0
Extensive alveolar consolidation	4	66.7	3	50.0	1	16.7
Oedema fluid in alveolar or bronchus	2	33.3	2	33.3	5	83.3

Gerbils infected with different doses of EV71 showed different pathology changes. In the high dose groups (1×10^5.5^ TCID50), as much as 90% of the lung tissue had been destroyed, with multiple foci of interstitial inflammatory cell infiltration, alveolar septum broadening, alveolar consolidation and local pulmonary hemorrhage observed (Fig. 3H). The main lesion characteristic was pulmonary interstitial inflammation. The most severe lung lesion was extensive pulmonary hemorrhaging in 37.5% (3/8) of EV71-infected gerbils (Fig. 3I). In the low dose groups (1×10^3.5^ TCID_50_), only 10–20% of the lung tissue had histological changes and no extensive pulmonary hemorrhaging was observed (Table 2). There were no obvious differences in histological examination between the IP and IM injected route. Evidence of very few areas of pulmonary interstitial thickening and alveolar septum broadening was found in the OL route groups. No local or extensive pulmonary hemorrhage was found (Table 3).

**Table 2:** The pathological types of lungs in EV71-infected gerbils with different virus doses.

Pathological findings	Virus titer 1×10^5.5^ (n = 8)		Virus titer 1×10^3.5^ (n = 8)	
	n	%	n	%
Pulmonary interstitial inflammatory cell infiltration	8	100.0	6	75.0
Alveolar septum broadening	8	100.0	6	75.0
Local alveolar consolidation	5	62.5	2	25.0
Interstitial pulmonary congestion	5	62.5	4	50.0
Extensive pulmonary hemorrhage	3	37.5	0	0
Extensive alveolar consolidation	1	12.5	0	0
Oedema fluid in alveolar or bronchus	5	62.5	5	62.5

21-day-old gerbils were inoculated EV71 by IP route.

**Table 3:** The pathological types of lungs in EV71-infected gerbils via different routes.

Pathological findings	IP (n = 8)		IM (n = 8)		OL (n = 8)	
	n	%	n	%	n	%
Pulmonary interstitial inflammatory cell infiltration	8	100.0	8	100.0	4	50.0
Alveolar septum broadening	8	100.0	8	100.0	4	50.0
Local alveolar consolidation	3	37.5	5	62.5	1	12.5
Interstitial pulmonary congestion	7	87.5	8	100.0	3	37.5
Extensive pulmonary hemorrhage	3	37.5	4	50	0	0
Extensive alveolar consolidation	4	50.0	3	37.5	0	0
Oedema fluid in alveolar or bronchus	7	87.5	7	87.5	3	37.5

21-day-old gerbils were inoculated with 1×10^5.5^ TCID_50_ EV71.

### Virus replication in organs of infected gerbils

**Fig 4:**
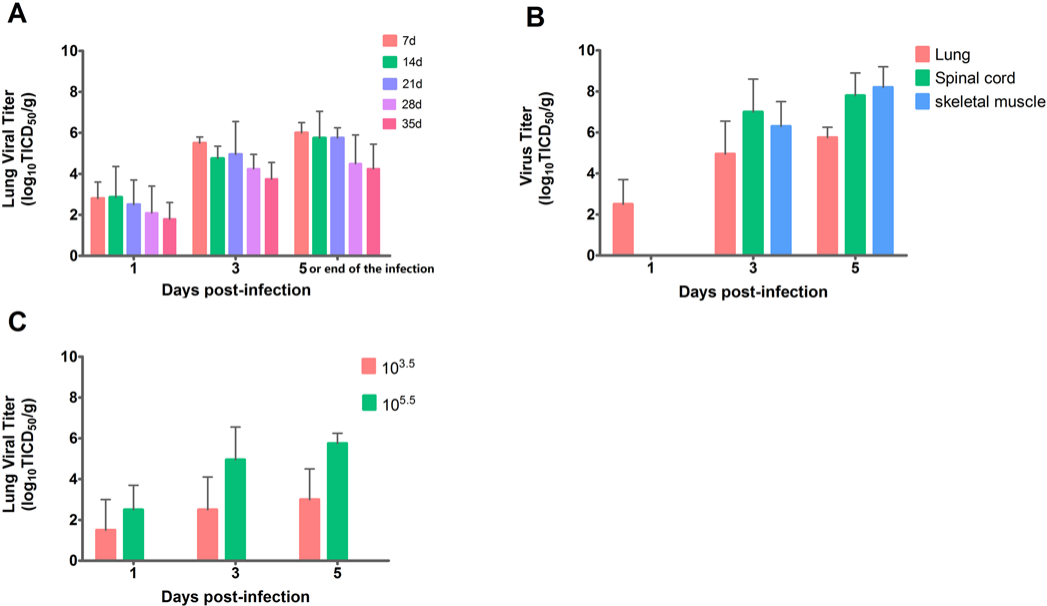
Virus replication in lung and other tissues of EV71-infected gerbils. (A) gerbils (7, 14, 21, 28, 35 d old) were inoculated IP with 1×10^5.5^ TCID_50_ of EV71 (n = 6 for each age groups). (B) Virus titer in various tissues was determined in 21-day-old gerbils inoculated with 1×10^5.5^ TCID_50_ of EV71 via IP route (n = 8 for each dose group). (C) 21-day-old gerbils were inoculated with 1×10^3.5^ or 1×10^5.5^ TCID_50_ of EV71 via IP.

We examinated the viral replication in gerbils infected with a dose of 1×10^5.5^ TCID_50_ by IP route in animals of five different ages. Virus isolated from the lung was detectable as early as one day p.i. with viral titers almost the same for 7–21 day-old gerbils. Further, this number was lower for 28–35 day old gerbils. Thereafter, virus titers increased gradually and reached a peak at the end of stage of EV71-infection in five age groups (Fig. 4A). In other tissues, such as liver, kidney, pancreas, stomach and spleen, virus replicated to the same level as that in lung tissues. The peak virus titers in the lung (1×10^5.75^ TCID_50_) were lower than that in the spinal cord, brainstem or skeletal muscle, the location of the highest observed virus titer 1×10^7.5~8.25^ TCID_50_ (Fig. 4B). Therefore, lung tissue was not the major target organ of EV71 infection in young gerbils. These data indicate that EV71 is a CNS- and muscle-tropic virus.

Peak virus titer was observed approximately one to two days later when infected with lower doses of EV71 (1×10^3.5^ TCID_50_). Further, the peak titer only reached 1×10^3.25^ TCID_50_; approximately two logs lower than those infected with high doses (Fig. 4C). In three asymptomatic gerbils infected with lower doses of EV71, no virus was detected in the lung tissues of two gerbils. These data suggest disease progression is dose dependent.

### EV71-specific antibodies in gerbils

**Fig 5:**
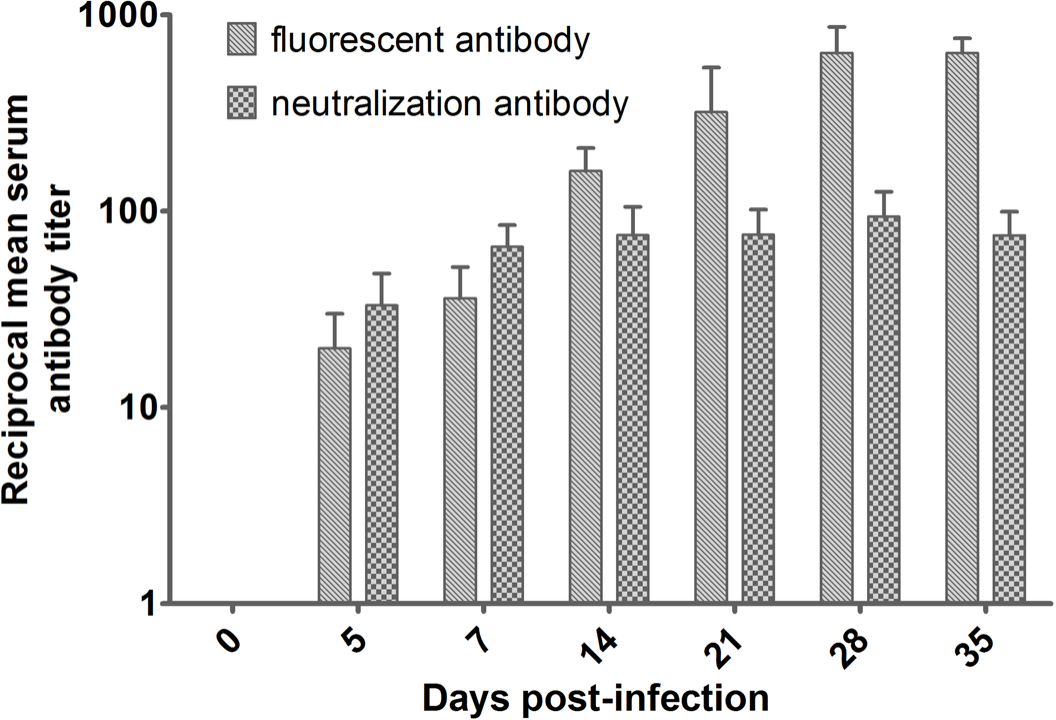
The reciprocal neutralizing antibody and fluorescent antibody titers of EV71-specific serum in infected gerbils following IP inoculation with 1×10^5.5^ TCID_50_ EV71. The solid line indicates mean antibody titer from three gerbils per group on a logarithmic scale for each day. Error bars represent the standard deviation.

Considering 7, 14, and 21 day-old gerbils infected via the IP route, all gerbils died before antibodies could be detected, we used 50-day-old gerbils for antibody detection. Eight gerbils were inoculated via the IP route with 1×10^5.5^ TCID_50_ EV71, and neutralizing antibodies were detected as early as day five days p.i. and peaked at 28 days p.i. at a ratio of 1:75.39 (Fig. 5).

### Protection against lung lesions in gerbils by passive immunization

**Fig 6:**
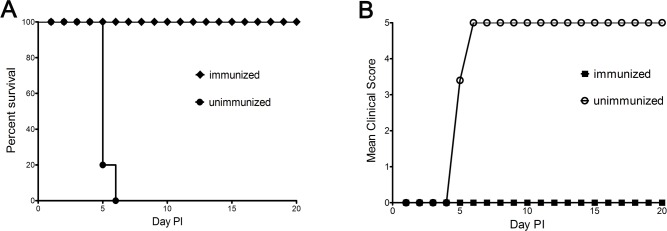
Survival and clinical score of immunized gerbils after IP challenge with humane doses of EV71 (100×HD_50_). Experiment groups (n = 5) were immunized with anti-EV71 antisera at 21-days-old as described in Materials and Methods. The control groups (n = 5) were given normal saline. All gerbils were challenged by the IP route with lethal doses of EV71 at post-immunization. The survival rates and clinical scores were monitored daily after challenge. *P* = 0.001.

**Fig 7:**
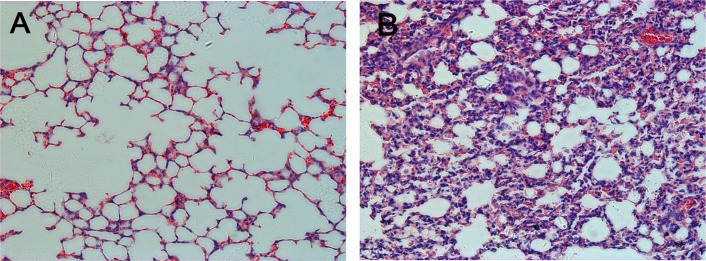
Histological examinations of lung in 21-day-old gerbils challenged with humane doses 100×HD_50_ EV71. (A) Gerbils receiving EV71-antisera revealed no evidence of inflammation and structures of the lungs appeared intact. (B) Gerbils receiving the mock sera, lung structures were damaged and multiple foci of pulmonary interstitial inflammatory were observed. Hematoxylin and eosin stain; magnification: A, B 200x.

Passive immunization was used to evaluate the effects of humoral immunity in protecting against EV71 lethal challenge. Gerbils were passively immunized with neutralizing antibodies (GMT = 89; 100 μL/gerbil). Two doses fully protected gerbils from EV71 lethal challenge. In contrast, mock-immunized gerbils developed hind limb paralysis and were dead at 5–6 days post-challenge (Fig. 6). Histological examination indicted gerbils that received the anti-EV71 antibodies had intact lung structures without detected EV71-induced lesions and no virus copies were detected in lungs by real-time RT-PCR. Mock-immunized gerbils revealed severe lung pathology including pulmonary interstitial inflammatory infiltration and alveolar septum broadening (Fig. 7). These results demonstrate passively acquired protection against EV71-induced lung lesions in the gerbil model is mediated by specific antisera containing neutralizing antibodies to EV71.

## Discussion

This study describes a promising animal model for the study of EV71 infection. Specifically, this experimental gerbil model is the first to demonstrate severe pulmonary lesions upon infection with this virus. We examined histopathological changes and virus replication in lung tissues of EV71-infected gerbils. Our results indicated that gerbils were susceptible to EV71 infection and the resulting severe pulmonary lesions when inoculated via IM or IP routes within the suckling periods (first 21 days of life). Symptoms such as interstitial pneumonia, pulmonary congestion and extensive lung hemorrhaging correlated with virus replication in the host animal lungs. Importantly, we demonstrated a protective role for passive antibody treatment in preventing disease in the gerbil model.

Many animal models have been used to study EV71 infection. Fourteen-day-old ICR mice, human SCARB2 transgenic mice and 14-day-old AG129 mice lacking IFN-α/β and IFN-γ receptor could mimic some neurologic complications typically observed in human EV71 infection [20,28–30]. The non-human primate model is much more similar to humans, however, only 10% of EV71-infected cynomolgus monkeys developed neurological disease[31]. Therefore, pulmonary lesion had not been reflected in these animal models which limits the applicability for understanding EV71 disease mechanisms [32]. In this study, we found gerbils developed unique severe pulmonary lesions after EV71 infection via IP or IM routes and the disease progressed rapidly with death occurring within 12 hours of first observing symptoms. Severe histopathological changes, including interstitial pneumonia, pulmonary lesions and extensive hemorrhaging, were observed in the lung tissue. We hypothesized that the presence of pulmonary lesions or hemorrhaging contributed to such a rapid progression of disease. Gerbil models that developed severe pulmonary lesions with rapid disease progression are compared to symptoms in humans known as fulminant pulmonary edema following EV71 infection [3,33]. Severe neurological disorders were also observed in a gerbil model in our previous study [26]. Therefore, gerbil animal models developed both neurological and respiratory clinical features consistent with those observed in humans.

Pulmonary edema is associated with fatal EV71 infection in children [3,4,15,34,35]. Histopathological examination of lung tissue reveals severe pulmonary infiltration and hemorrhagic edema [36,37]. Wu et al. suggested that brainstem lesions and medullar damage could induce autonomic nervous dysfunction, in turn altering pulmonary vascular permeability eventually resulting in pulmonary edema [33]. Chang et al. revealed systemic hypertension and vasoconstriction produced by central sympathetic over activation was responsible for pulmonary edema in humans [3]. While brainstem lesions appeared in cynomolgus monkeys, ICR mice and AG129 mice, no animals developed pulmonary edema. Otherwise, EV71 replication in lungs was boosted at early stages of infection and decreased to a very low level at later time points post-infection in AG129 mice [20,28]. In contrast, EV71 infected gerbil lung tissue and viral replication was persistent, with viral titers reaching 1×10^5.75^ TCID_50_, until they died. We hypothesized that the damage associated with virus replication and the stimulation of inflammatory cytokines in lung tissues is a possible mechanism of pulmonary edema in gerbils. Since the pathogenesis of EV71-induced pulmonary edema and hemorrhage remains unclear, we hypothesized that this gerbil model would provide a useful platform for understanding the pathogenesis of pulmonary edema.

Skeletal muscle has persistent EV71 viral titers and is a vigorous viral source for entry into the blood stream or the CNS, sometimes via peripheral motor nerves [21,38]. These studies suggest IM inoculation of EV71 should be more fatal than IP inoculation, however, we found disease progression to be similar between the two routes in gerbils. In fact, when infected with the same EV71 dose, each inoculation route resulted in death at the same time post infection. Moreover, virus in the muscle tissue was not detected until 3 days p.i. following IP inoculation, suggesting the bloodstream may be the major route of spread in the gerbil model with EV71 spread from muscle to CNS through neuronal pathways constituting a secondary pathway.

Fecal-oral transmission is typically thought to be the main route of EV71 spread, but evidence to support this transmission mode was limited to a few studies [39,40]. ICR pups did not transmit EV71 to their cagemates or dam by clinical isolated EV71[41], showing this model is not ideal for the study of EV71 infection. EV71 has been isolated in stool specimens and respiratory secretions of EV71 infected children [42], therefore EV71 transmission more than likely occurs by both respiratory and fecal-oral spread. In our preview study, we found EV71 could transmit from infected gerbil to their cagemates on the basis of seroconversion by the field virus strain (data not shown). Here, we demonstrated EV71 could infect gerbils by IM, IP, OL and even by nose route (data not shown). Therefore, EV71 has the ability to infect by multiple routes in a gerbil model, suggesting EV71 infection may transmit by multiple routes in humans. Gerbils may provide an ideal animal model for further investigating various transmission routes infected by EV71. Otherwise, humans are the only known natural host of EV71. Gerbils were so sensitive to EV71 infection and it implied a properly potential reservoir host for EV71 viruses. These findings expanded our view on natural reservoirs of EV71.

Passive immunization fully protected animals from EV71-induced death in newborn mice[41]. Our results here further confirmed the protective role of passive antibodies in EV71 infection in a gerbil model. Kinetic experiments showed that neutralizing antibodies can be detected at 5–7 days after infection and peak antibody titers appeared at 28 days with the duration of high neutralizing antibody production lasting as long as one month. These results implied that passive immunization against EV71 might be an important alternative therapeutic choice for patients with early EV71 infection.

In conclusion, studies in animal models are essential for understanding the pathophysiology of EV71 and developing therapeutic strategies after EV71 infection. Based on histopathological analysis, our data showed that gerbils can be effectively infected by EV71 and exhibited both neurological and respiratory symptoms in line with those observed in EV71-infected humans. We suggest the gerbil model is the most appropriate model for studying EV71 human disease. EV71-infection induced various pulmonary damage levels in gerbils providing a powerful tool to study the host-pathogen interface of EV71 infection. Finally, the gerbil model can serve as a valuable model for the development of vaccines and therapeutics in future.

## Supporting Information

S1 Table Gerbils were inoculated IP with 1×10^5.5^ TCID_50_ of EV71 at the age of 7 days. (DOCX)

S2 Table Gerbils were inoculated IP with 1×10^5.5^ TCID_50_ of EV71 at the age of 14 days. (DOCX)

S3 Table Gerbils were inoculated IP with 1×10^5.5^ TCID_50_ of EV71 at the age of 21 days. (DOCX)

S4 Table Gerbils were inoculated IP with 1×10^5.5^ TCID_50_ of EV71 at the age of 28 days. (DOCX)

S5 Table Gerbils were inoculated IP with 1×10^5.5^ TCID_50_ of EV71 at the age of 35 days. (DOCX)

S6 Table 21-day-old gerbils were inoculated with 1×10^3.5^ or 1×10^5.5^ TCID_50_ of EV71 via IP. (DOCX)

S7 Table 21-day-old gerbils were inoculated with 1×10^5.5^ TCID_50_ of EV71 via IP, IM or OL routes. (DOCX)
