# Supplementary material for: Enterovirus 71 Infection Causes Severe Pulmonary Lesions in Gerbils, Meriones unguiculatus, Which Can Be Prevented by Passive Immunization with Specific Antisera
Source: PLoS One. 2015 Mar 13;10(3):e0119173. doi: 10.1371/journal.pone.0119173 (PMC4359154; doi:10.1371/journal.pone.0119173)
Supplement: S1 Table — (DOCX) [file pone.0119173.s001.docx]

**Table S1. Gerbils were inoculated IP with 1×10^5.5^ TCID_50_ of EV71 at the age of 7 days.**

| Days post-infection | 7d gerbils(n=6) | |
| --- | --- | --- |
|  | Weigh(g) ±SD | Status |
| 0 | 8.18±0.54 | Health:6 |
| 1 | 8.56±0.53 | Health:6 |
| 2 | 8.91±0.73 | Health:6 |
| 3 | 9.25±0.73 | Death:1; lethargy:3(euthanased); shallow breathing:1; tachypnea:2 |
| 4 | 8.57±0.78 | Death:1; lethargy:1(euthanased); |
