# Supplementary material for: Enterovirus 71 Infection Causes Severe Pulmonary Lesions in Gerbils, Meriones unguiculatus, Which Can Be Prevented by Passive Immunization with Specific Antisera
Source: PLoS One. 2015 Mar 13;10(3):e0119173. doi: 10.1371/journal.pone.0119173 (PMC4359154; doi:10.1371/journal.pone.0119173)
Supplement: S2 Table — (DOCX) [file pone.0119173.s002.docx]

**Table S2. Gerbils were inoculated IP with 1×10^5.5^ TCID_50_ of EV71 at the age of 14 days.**

| Days post-infection | 14d gerbils(n=6) | |
| --- | --- | --- |
|  | Weigh(g) ±SD | Status |
| 0 | 12.64±0.70 | Health:6 |
| 1 | 13.36±0.79 | Health:6 |
| 2 | 13.77±0.79 | Health:6 |
| 3 | 14.08±0.85 | Health:4; limb weakness:2 |
| 4 | 13.61±0.92 | Death:2; lethargy:3(euthanased); tachypnea:2; respiratory distress:2; 1 hind limb paralysis:3 |
| 5 | 13.25 | Death:1 |
