# Supplementary material for: Enterovirus 71 Infection Causes Severe Pulmonary Lesions in Gerbils, Meriones unguiculatus, Which Can Be Prevented by Passive Immunization with Specific Antisera
Source: PLoS One. 2015 Mar 13;10(3):e0119173. doi: 10.1371/journal.pone.0119173 (PMC4359154; doi:10.1371/journal.pone.0119173)
Supplement: S3 Table — (DOCX) [file pone.0119173.s003.docx]

**Table S3. Gerbils were inoculated IP with 1×10^5.5^ TCID_50_ of EV71 at the age of 21 days.**

| Days post-infection | 21d gerbils(n=6) | |
| --- | --- | --- |
|  | Weigh(g) ±SD | Status |
| 0 | 15.32±1.46 | Health:6 |
| 1 | 15.82±2.02 | Health:6 |
| 2 | 16.18±1.82 | Health:6 |
| 3 | 17.65±1.46 | Health:6 |
| 4 | 16.71±1.69 | 1 hind limb paralysis:6 |
| 5 | 16.56±1.89 | 2 hind limb paralysis:3; lethargy:4(euthanased); tachypnea:2;shallow breathing:2 |
| 6 | 17.2±1.95 | Death:2 |
