# Supplementary material for: Enterovirus 71 Infection Causes Severe Pulmonary Lesions in Gerbils, Meriones unguiculatus, Which Can Be Prevented by Passive Immunization with Specific Antisera
Source: PLoS One. 2015 Mar 13;10(3):e0119173. doi: 10.1371/journal.pone.0119173 (PMC4359154; doi:10.1371/journal.pone.0119173)
Supplement: S4 Table — (DOCX) [file pone.0119173.s004.docx]

**Table S4. Gerbils were inoculated IP with 1×10^5.5^ TCID_50_ of EV71 at the age of 28 days.**

| Days post-infection | 28d gerbils(n=6) | |
| --- | --- | --- |
|  | Weigh(g) ±SD | Status |
| 0 | 19.86±0.78 | Health:6 |
| 1 | 20.81±0.78 | Health:6 |
| 2 | 22.56±0.89 | Health:6 |
| 3 | 23.61±0.94 | Health:6 |
| 4 | 24.17±1.26 | Health:6 |
| 5 | 24.84±1.93 | Reduced mobility:2 |
| 6 | 25.32±1.67 | 1 hind limb paralysis:1; limb weakness:2 |
| 7 | 27.83±2.32 | Death:2 |
| 11 | 29.56±1.39 | Health:1;1 hind limb paralysis:1; limb weakness:2 |
| 14 | 33.47±2.26 | Health:3;1 hind limb paralysis:1 |
| 20 | 41.27±3.15 | Health:3;1 hind limb paralysis:1 |
