# Supplementary material for: Enterovirus 71 Infection Causes Severe Pulmonary Lesions in Gerbils, Meriones unguiculatus, Which Can Be Prevented by Passive Immunization with Specific Antisera
Source: PLoS One. 2015 Mar 13;10(3):e0119173. doi: 10.1371/journal.pone.0119173 (PMC4359154; doi:10.1371/journal.pone.0119173)
Supplement: S5 Table — (DOCX) [file pone.0119173.s005.docx]

**Table S5.** **Gerbils were inoculated IP with 1×10^5.5^ TCID_50_ of EV71 at the age of 35 days.**

| Days post-infection | 35d gerbils(n=6) | |
| --- | --- | --- |
|  | Weigh(g) ±SD | Status |
| 0 | 28.83±0.40 | Health:6 |
| 1 | 31.35±0.58 | Health:6 |
| 2 | 32.40±1.03 | Health:6 |
| 3 | 34.59±0.91 | Health:6 |
| 4 | 35.75±1.14 | Health:6 |
| 5 | 36.35±1.49 | Health:6 |
| 6 | 37.43±1.44 | Health:6 |
| 7 | 37.92±1.16 | Health:5;1 hind limb paralysis:1 |
| 8 | 38.68±1.82 | Health:4;1 hind limb paralysis:2 |
| 14 | 43.07±1.94 | Health:4;1 hind limb paralysis:2 |
| 20 | 57.23±2.15 | Health:5; 1 hind limb paralysis:1 |
