# Supplementary material for: Enterovirus 71 Infection Causes Severe Pulmonary Lesions in Gerbils, Meriones unguiculatus, Which Can Be Prevented by Passive Immunization with Specific Antisera
Source: PLoS One. 2015 Mar 13;10(3):e0119173. doi: 10.1371/journal.pone.0119173 (PMC4359154; doi:10.1371/journal.pone.0119173)
Supplement: S6 Table — (DOCX) [file pone.0119173.s006.docx]

**Table S6. 21-day-old gerbils were inoculated with 1×10^3.5^ or 1×10^5.5^ TCID_50_ of EV71 via IP.**

| Days post-infection | 21d gerbils(n=8) | | 21d gerbils(n=8) | | 21d gerbils(n=5) | |
| --- | --- | --- | --- | --- | --- | --- |
|  | 1×10^3.5^ | | 1×10^5.5^ | | control | |
|  | Weigh(g) ±SD | Status | Weigh(g) ±SD | Status | Weigh(g) ±SD | Status |
| 0 | 15.62±0.63 | Health:8 | 14.43±0.50 | Health:8 | 15.42±1.56 | Health:5 |
| 1 | 15.82±0.79 | Health:8 | 15.17±0.63 | Health:8 | 15.89±1.64 | Health:5 |
| 2 | 16.12±0.54 | Health:8 | 15.69±0.74 | Health:8 | 16.34±1.76 | Health:5 |
| 3 | 16.35±0.86 | Health:8 | 16.25±0.77 | Health:8 | 17.27±1.35 | Health:5 |
| 4 | 17.04±1.28 | Health:8 | 16.08±0.82 | 1 hind limb paralysis:1 | 17.58±1.78 | Health:5 |
| 5 | 16.91±0.93 | Health:8 | 15.62±0.66 | Death:2; 2 hind limb paralysis:2(euthanased); tachypnea:3;lethargy:1(euthanased) | 18.05±2.17 | Health:5 |
| 6 | 17.83±1.03 | Health:8 | 15.68±0.85 | Death:2; 1 hind limb paralysis:1 | 18.90±1.69 | Health:5 |
| 7 | 18.28±1.49 | Health:3;2 hind limb paralysis:2(euthanased);  1 hind limb paralysis:3;tachypnea:2 | 16.68 | Death:1 | 19.05±1.91 | Health:5 |
| 8 | 18.13±1.33 | Health:3; 1 hind limb paralysis:3 |  |  | 20.75±2.27 | Health:5 |
| 9 | 18.76±1.78 | Health:3;death:1; 1 hind limb paralysis:2 |  |  | 21.82±2.05 | Health:5 |
| 10 | 19.56±1.84 | Health:3;1 hind limb paralysis:2 |  |  | 22.16±2.36 | Health:5 |
| 20 | 33.47±2.56 | Health:3;1 hind limb paralysis:2 |  |  | 35.65±2.48 | Health:5 |
