# Supplementary material for: Enterovirus 71 Infection Causes Severe Pulmonary Lesions in Gerbils, Meriones unguiculatus, Which Can Be Prevented by Passive Immunization with Specific Antisera
Source: PLoS One. 2015 Mar 13;10(3):e0119173. doi: 10.1371/journal.pone.0119173 (PMC4359154; doi:10.1371/journal.pone.0119173)
Supplement: S7 Table — (DOCX) [file pone.0119173.s007.docx]

**Table S7.** **21-day-old gerbils were inoculated with 1×10^5.5^ TCID_50_ of EV71 via IP, IM or OL routes.**

| Days post-infection | 21d gerbils(n=8) | | 21d gerbils(n=8) | | 21d gerbils(n=8) | |
| --- | --- | --- | --- | --- | --- | --- |
|  | IP | | IM | | OL | |
|  | Weigh(g) ±SD | Status | Weigh(g) ±SD | Status | Weigh(g) ±SD | Status |
| 0 | 15.00±0.45 | Health:8 | 14.85±1.67 | Health:8 | 15.86±0.83 | Health:8 |
| 1 | 15.53±0.63 | Health:8 | 15.65±1.45 | Health:8 | 16.32±0.65 | Health:8 |
| 2 | 15.89±1.31 | Health:8 | 15.74±1.33 | Health:8 | 16.43±0.73 | Health:8 |
| 3 | 16.48±0.52 | Health:8 | 16.98±1.66 | Health:8 | 17.38±0.46 | Health:8 |
| 4 | 17.04±0.65 | Health:6; limb weakness:2 | 17.54±1.96 | Health:8 | 17.93±0.35 | Health:8 |
| 5 | 17.45±0.83 | Death:5; tachypnea:2; 1 hind limb paralysis:1 | 18.18±1.75 | Death:5; shallow breathing:2; 1 hind limb paralysis:3 | 18.82±0.64 | Health:8 |
| 6 | 18.94±0.56 | Death:2; 1 hind limb paralysis:1; tachypnea:1 | 18.32±1.49 | Death:3; | 19.76±0.78 | Health:8 |
| 7 | 18.47 | Death:1; |  |  | 20.82±0.85 | Health:8 |
| 8 |  |  |  |  | 20.47±0.59 | Health:8 |
| 9 |  |  |  |  | 21.18±0.77 | Health:8 |
| 10 |  |  |  |  | 21.98±0.84 | health:6;limb weakness:2 |
| 11 |  |  |  |  | 22.35±1.23 | health:6;Death:1; limb weakness:1 |
| 12 |  |  |  |  | 22.50±1.04 | Health:6; limb weakness:1 |
| 20 |  |  |  |  | 34.74±1.26 | Health:6; limb weakness:1 |
